# Simple Scaling as a Tool to Help Assess the Closure-Free *da*/*dN* Versus Δ*K_eff_* Curve in a Range of Materials

**DOI:** 10.3390/ma17225423

**Published:** 2024-11-06

**Authors:** Rhys Jones, Andrew S. M. Ang, Daren Peng

**Affiliations:** 1 Centre of Expertise for Structural Mechanics, Department of Mechanical and Aerospace Engineering, Monash University, Clayton, VIC 3800, Australiadaren.peng@monash.edu (D.P.); 2 ARC Industrial Transformation Training Centre on Surface Engineering for Advanced Materials, Faculty of Science, Engineering and Technology, Swinburne University of Technology, John Street, Hawthorn, VIC 3122, Australia

**Keywords:** simple scaling, crack closure, fatigue crack growth, Ti-6Al-4V ELI, ASTM E647-23b

## Abstract

Recent studies have proposed a simple formula, which is based on Elber’s original approach to account for *R*-ratio effects, for determining the crack closure-free Δ*K_eff_* versus *da*/*dN* curve from the measured *R*-ratio-dependent Δ*K* versus *da*/*dN* curves. This approach, which is termed “Simple Scaling,” has been shown to collapse the various *R*-ratio-dependent curves onto a single curve. Indeed, this approach has been verified for a number of tests on metals, polymers, and a medium-entropy alloy. However, it has not yet been used to help assess/determine the closure-free Δ*K_eff_* versus *da*/*dN* curve. The current paper addresses this shortcoming and illustrates how to use this methodology to assess the Δ*K_eff_* versus *da*/*dN* curves given in the open literature for tests on a number of steels, aluminum alloys, STOA Ti-6Al-4V, a magnesium alloy, and Rene 95. As such, it would appear to be a useful tool for assessing fatigue crack growth.

## 1. Introduction

It has long been known that the Δ*K* versus *da*/*dN* curves measured using ASTM E647-23b [1] fatigue standard tests on long cracks can be *R*-ratio dependent. (Here, *K* is the stress intensity factor; *K_min_* and *K_max_* are the minimum and maximum values of *K* in a load cycle, respectively; Δ*K* = *K_max_* − *K_min_*; *R* = *K_min_*/*K_max_*; *N* is the number of cycles; and *a* is the crack length). To account for this effect, Elber [2,3] introduced the concept of an effective stress intensity factor (Δ*K_eff_*), which is defined as follows:

Δ*K_eff_* = *U*(*R*) Δ*K* (1)

where the function *U*(*R*) is chosen such that, for long cracks that experience plastic wake-induced crack closure, the resultant Δ*K_eff_* versus *da*/*dN* curves all fall onto a single curve regardless of the *R*-ratio. Equation (1) is also commonly written as follows:

Δ*K_eff_* = (*K_max_* − *K*_o_) = *U*(*R*) Δ*K* (2)

where *K*_o_ is defined as the value of stress intensity factor at which the crack first opens. As such, the equation for *K*_o_ is expressed as follows:

*K*_o_*= K_max_* (1 − *U*(*R*) × *R*) (3)

It follows from Equation (3) that the general formula for the load at which a crack opens, which we will define as *P*_o_, is given by:

*P*_o_*= P_max_* (1 − *U*(*R*) × *R*) (4)

where *P_max_* is the maximum load in the cycle.

As noted by Paris et al. [4], Paris et al. [5], Schijve [6], and Jones [7], crack closure approaches are now widely used to assess the damage tolerance of aerospace components, and numerous formulae for determining the function *U*(*R*) and hence the *R*-ratio-independent Δ*K_eff_* versus *da*/*dN* curve have been proposed (see [2,3,4,5,6,8,9,10,11,12,13,14]). As a result, methods for determining Δ*K*_eff_ can now be found in the fatigue test standard ASTM E647-23b. The methods discussed in ASTM E647-23b [1] include determining the “crack opening force” from compliance measurements and using the adjusted compliance ratio (ACR) approach, which was first developed by Donald et al. [15] and is discussed in more detail in the paper by Donald and Paris [16]. These approaches are discussed in ASTM E647-23b Appendices X2 and X4 [1], respectively. (Despite this, as mentioned by Kujawski et al. [17] and Gonzalez et al. [18], there is still a debate about the physics associated with crack closure and the concept of a crack opening force).

It should also be noted that the test protocol outlined in Sections 8.5 and 8.6 of the fatigue test standard ASTM E647-23b [1] recommends that, for *da*/*dN* ≤ 10^−8^ m/cycle, load reduction approaches are used to determine the crack growth curve. For *da*/*dN* ≥ 10^−8^ m/cycle, the “constant-force amplitude” test procedure is recommended. In this approach, the amplitude of the load Δ*P* (=*P_max_* − *P_min_*), where *P_min_* and *P_max_* are the minimum and maximum loads in a cycle, respectively, is kept constant throughout the test. This results in Δ*K* increasing as the crack grows. However, Section 8.7 also allows for tests where *K_max_* is kept constant and notes that this test procedure can help to determine more conservative values of the near threshold behavior. It should also be noted that, for tests on long cracks, ASTM E647-23b requires the test specimens to be pre-cracked. In the context of this paper, it should also be noted that compression pre-cracking constant amplitude (CPCA) followed by load reduction (CPLR) methods are now often used to generate the Δ*K* versus *da*/*dN* curves for *da*/*dN* ≤ 10^−8^ m/cycle (see [19,20,21]). Furthermore, as in [19,20], a back face strain gauge is also sometimes used to monitor both the crack length and the crack opening. In this context, it should also be noted that Gonzales et al. [18] revealed that such back face strain gauge measurements do not necessarily reflect the actual (true) crack opening load. The paper by Schönbauer et al. [22] revealed that, for 17-4PH steel, *R*-ratio effects disappeared when *da*/*dN* was plotted as a function of Δ*K*/Δ*K_th_.* Here, Δ*K_th_* is the ASTM-defined fatigue threshold, i.e., the value of Δ*K* at *da*/*dN* = 10^−10^ m/cycle. This observation was subsequently supported by the results presented in [23,24] for the growth of cracks in a range of metals and a medium-entropy alloy:

- Aerospace aluminum alloys 2024-T3, 7075-T651, 7050-T7451, 6013-T651, 2324-T39, 7085-T7452, 2524-T3, and 7049;
- 304L stainless steel;
- Titanium alloy Ti622;
- Selective laser melt (SLM)-built Inconel 625 and the Chinese super alloy GH4169;
- Laser powder bed fusion (LPBF)-built Hastelloy X;
- Medium-entropy cantor alloy CrCoNi.

Reference [24] also revealed that, from a mathematical standpoint, *U*(*R*) can be expressed as follows:

*U*(*R*) = Δ*K*_*eff,da*/*dN*_/Δ*K*_*da*/*dN*_(R) (5)

where Δ*K_eff,da_*_/*dN*_, and Δ*K_da_*_/*dN*_(*R*) are the values of Δ*K_eff_* and Δ*K*(*R*) at a given (low) value of *da*/*dN*, respectively. (Various alternative formulae for the function *U*(*R*) can be found in [4,5,6,7,8,9,10,11,12,13,14,15,16,18]). Reference [24] also noted that what this means is that, for metals that conform to Elber’s original hypothesis, *R*-ratio effects are merely a reflection of the change in the fatigue threshold and that, for metals that conform to Elber’s original hypothesis, the Δ*K*/Δ*K_th_* versus *da*/*dN* curves should be independent of the *R*-ratio. Furthermore, ref. [24] also found that there were several instances when expressing *da*/*dN* as a function of Δ*K*/Δ*K_th_* was able to reveal that the effect of temperature on the Δ*K* versus *da*/*dN* curves was also (to a first approximation) merely a reflection of the change in the fatigue threshold due to the change in temperature. The subsequent paper [25] extended these observations to account for the effect of different molecular weights on the growth of cracks in high-density polyethene and to understand the effect of different specimen geometries, *R*-ratios, and different levels of irradiation on crack growth in high-density polyethene.

Whereas ref. [24] focused on *R*-ratio effects in metals and high-entropy alloys and ref. [25] focused on crack growth in high-density polyethene, refs. [23,26,27,28,29,30] addressed the problem of delamination growth in composites. References [23,26,27,28,29,30] revealed that the Δ√*G* versus *da*/*dN* curves, where *G* is the energy release rate associated with delamination growth in composites, were often a strong function of the length of the pre-crack prior to testing and that this effect was due to retardation effects due to fiber bridging. Papers [23,26,27,28,29,30] also reported that expressing *da*/*dN* as a function of Δ√*G*/Δ√*Gth* also often collapsed the various pre-crack length-dependent Δ√*G* versus *da*/*dN* curves onto a single curve. As such, these various observations helped to clarify the fracture mechanics parameters associated with different test procedures, test temperatures, different levels of irradiation, and a range of non-metallic materials, both polymers and fiber composite laminates. As such, the Simple Scaling variant of Elber’s original hypothesis would appear to have the advantage over other approaches in that can help clarify the underlying parameters for a wide class of problems and for tests where, whilst the *R*-ratio is fixed, the test conditions are different, for example, different test temperatures, different levels of irradiation, or (as in the case of delamination growth in composites) different levels of pre-cracking. However, to date, the Simple Scaling approach has not been used to help assess closure-free Δ*K_eff_* versus *da*/*dN* curves.

## 2. Materials and Methods

Reference [24] was the first to reveal that plotting *da*/*dN* in terms of non-dimensional ratio Δ*K*/Δ*K_th_* could be useful for assessing the fracture mechanics parameters governing the effects of both temperature and *R*-ratio on the Δ*K* versus *da*/*dN* crack growth curves. This was achieved by considering their effects on the curves associated with (i) 304L stainless steel, (ii) the Chinese super alloy GH4169, and (iii) the medium-entropy cantor alloy CrCoNi.

To further investigate this observation, we examine the Δ*K* versus *da*/*dN* curves presented in [31,32] for crack growth in titanium alloy Ti-6Al-4V ELI tested at −4 °K (−269 °C), 25 °C, and 250 °C. This study also reveals that when *da*/*dN* is expressed in terms of Δ*K*/Δ*K_th_*, then, to a first approximation, the different temperature-dependent Δ*K* versus *da*/*dN* curves essentially collapse onto a single curve that is independent of both the *R*-ratio and the test temperature. This finding further highlights the advantages of plotting *da*/*dN* in terms of the ratio Δ*K*/Δ*K_th_* and thereby further illustrates the usefulness of Equation (5) for assessing the effect of the test temperature on fatigue crack growth. This particular material, i.e., Ti-6Al-4AV ELI, was chosen since it is used in the F-35 Lightning II.

We next evaluate if Equation (5) can be used to rapidly assess, and possibly improve, the Δ*K_eff_* versus *da*/*dN* curves given in the open literature for a range of metals:

- The *R* = 0.1, 0.4, 0.7, 0.9, and 0.95 Δ*K* versus *da*/*dN* curves given by Newman [19] for the growth of long cracks in 9310 steel;
- The *R* = 0.8, 0.5, 0.3, and 0.1 Δ*K* versus *da*/*dN* curves given by Boyce and Ritchie [33] and the Δ*K* versus *da*/*dN* curve given by Newman, Vizzini, and Yamada [34] for STOA Ti-6Al-4V;
- The *R* = 0.1, 0.4, 0.7, 0.8, and 0.9 Δ*K* versus *da*/*dN* curves and the Δ*K_eff_* versus *da*/*dN* curve given in [35] for aluminum alloy 7075-T7351;
- The *R* = 0.7, 0.5, 0.3, 0.1, and −1.0 Δ*K* versus *da*/*dN* curves given by Donald and Lados [36] for aluminum alloy 2324-T39;
- The *R* = 0.7 and 0.1 Δ*K* versus *da*/*dN* curves and the corresponding Δ*K_eff_* versus *da*/*dN* curve given in [37] for aluminum alloy 7249-T6511;
- The *R* = 0.1 and 0.7 Δ*K* versus *da*/*dN* curves and the Δ*K_eff_* versus *da*/*dN* curves given by Newman, Vizzini, and Yamada [34] for magnesium alloy Mg AZ91E;
- The *R* = 0.95, 0.7, 0.4, and 0.1 Δ*K* versus *da*/*dN* curves and the Δ*K_eff_* versus *da*/*dN* curves given by Yamada and Newman [20] for crack growth in 4340 steel;
- The *R* = 0.5 and 0.1 Δ*K* versus *da*/*dN* curves and the corresponding Δ*K_eff_* versus *da*/*dN* curve given by Liaw et al. [38] for a Mn-Cr austenitic steel;
- The Δ*K* versus *da*/*dN* curves given by Newman and Piascik [39] for the super alloy Rene 95. These were *K_max_* tests with *K_max_* values of 22, 88, and 132 MPa √m.

Unless otherwise stated, the various Δ*K* versus *da*/*dN* tests studied in this paper were ATSTM E647-23b [1] standard compact tension (CT) specimens, ala ASTM test standard E647, and the tests were performed in accordance with the test protocol outlined in Sections 8.5–8.7 of the test standard ASTM E647-23b [1]. Furthermore, unless otherwise stated, all tests were performed at room temperature.

Whilst, in each case, it is shown that Equation (5) yields *da*/*dN* versus Δ*K_eff_* curves that can be quickly and easily obtained, there are instances, for example, when the estimates for the Δ*K_eff_* versus *da*/*dN* curves obtained by alternative methods do not coincide sufficiently, and it can help to resolve anomalies.

## 3. Crack Growth in Ti-6Al-4V over a Range of *R*-Ratios and Test Temperatures

**Figure 1:**
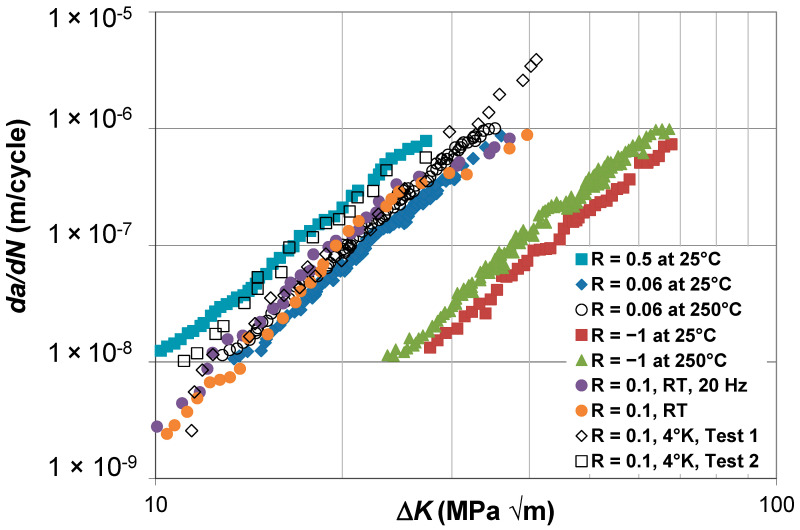
The Δ*K* versus *da*/*dN* curves for Ti-6Al-4V ELI tested at −4 °K (−269 °C), 25 °C, and 250 °C (The *da*/*dN* versus Δ*K* curves are adapted from [31,32]).

In the introduction, we highlighted that refs. [22,23,24,25] had revealed that this Simple Scaling approach would appear to have the advantage over other approaches for modeling crack closure and had the potential to help clarify the underlying parameters for a wide class of problems and for tests where, whilst the *R*-ratio is fixed, the test conditions are different, for example, different test temperatures, different levels of irradiation, or (as in the case of delamination growth in composites) different levels of pre-cracking. To further investigate this potential, let us examine the *R* = 0.5, 0.06, and -1 Δ*K* versus *da*/*dN* curves given in [31] for the growth of long cracks in titanium alloy Ti-6Al-4V ELI tested at 25 °C and 250 °C, which are reproduced in Figure 1. With the exception of the *R* = 0.1 room temperature test labeled “*R* = 0.1, RT, 20 Hz”, which was performed at a test frequency of 20 Hz, all tests were performed at a test frequency of 5 Hz. The *R* = 0.1 Δ*K* versus *da*/*dN* curves given in [32] for the growth of long cracks in titanium alloy Ti-6Al-4V ELI tested at room temperature and at −4 °K (−269 °C) are also reproduced in Figure 1. Figure 1 highlights that the Δ*K* versus *da*/*dN* curves have a strong dependency both on the *R*-ratio and the test temperature.

**Figure 2:**
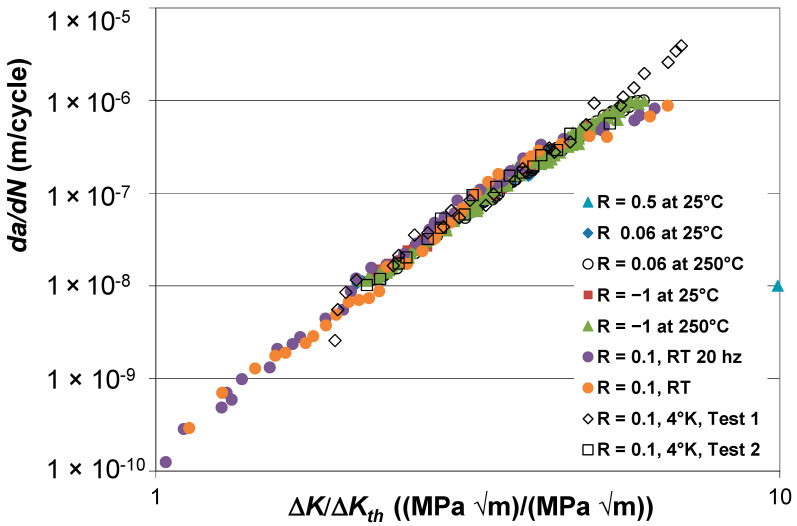
The Δ*K*/Δ*K_th_* versus *da*/*dN* curves for Ti-6Al-4V ELI tested at −4 °K (−269 °C), 25 °C, and 250 °C.

However, Figure 2 reveals that if *da*/*dN* is plotted against Δ*K*/Δ*K_th_*, then, to a first approximation, all of the different curves shown in Figure 1 now essentially collapse onto a curve that is independent of both the *R*-ratio and the test temperature. (Here, it should be noted that since not all of the curves had data down to a growth rate of 10^−10^ m/cycle, the value of Δ*K_t_*_h_ was chosen so as to make the various curves coincide at a crack growth rate of just over 10^−8^ m/cycle). The observation that, when plotted in this fashion, the different curves shown in Figure 1 essentially collapse suggests that, for Ti-6Al-4V ELI, both the test temperature and the *R*-ratio effects were primarily due to their effect on the fatigue threshold. This observation supports comments given in [24] on the insights that can be gained by plotting *da*/*dN* against Δ*K*/Δ*K_th_*. Whilst this phenomenon may not hold for other materials, it would nevertheless appear to support the statement given in the Introduction that there are clear advantages in taking the time to plot *da*/*dN* against Δ*K*/Δ*K_th_*. The values of used in Figure 2 are given in Table 1.

## 4. Crack Growth in 9310 Steel

**Figure 3:**
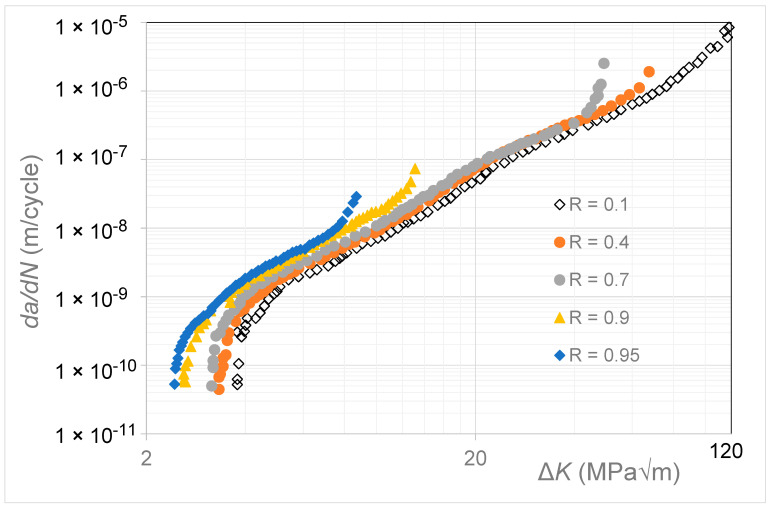
The Δ*K* versus *da*/*dN* curves for 9310 steel (Adapted from [19]).

The room temperature *R* = 0.1, 0.4, 0.7, 0.9, and 0.95 Δ*K* versus *da*/*dN* curves given by Newman [19] for the growth of long cracks in 9310 steel are reproduced in Figure 3. This study reported that, for *da*/*dN* ≤ 10^−8^ m/cycle, compression pre-cracking constant amplitude (CPCA) and load reduction (CPLR) methods were used to generate the Δ*K* versus *da*/*dN* curves. A back face strain gauge (BFS) gauge was used both to monitor crack growth and to determine the load at which the crack opened.

**Figure 4:**
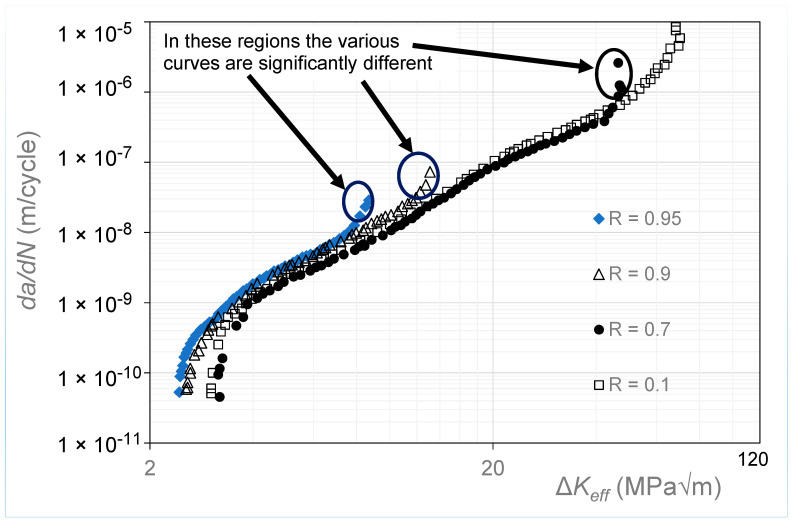
The Δ*K_eff_* versus *da*/*dN* curves given in [19] for 9310 steel (Adapted from [19]).

**Figure 5:**
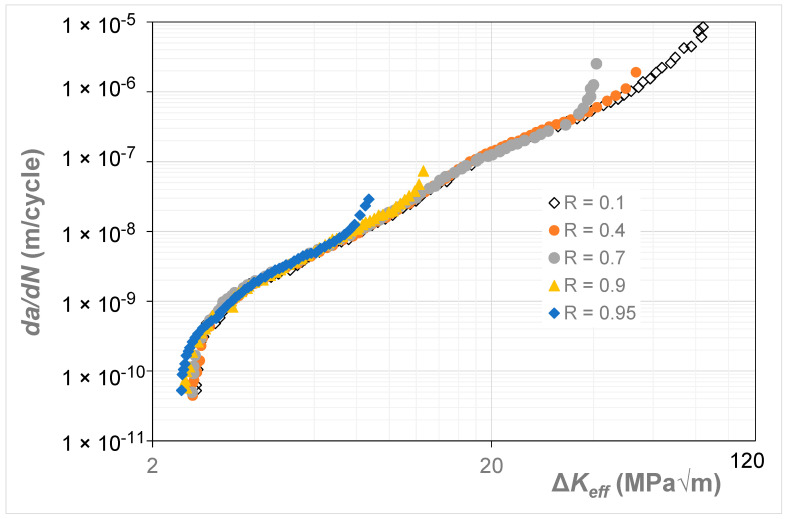
The Δ*K_eff_* versus *da*/*dN* curves for 9310 steel determined using Equation (5).

In [19], the Δ*K_eff_* versus *da*/*dN* curves were determined using the “analytical crack closure equation” given by Newman [10], with the constraint factor set to 2.5. The resultant Δ*K_eff_* versus *da*/*dN* curves are shown in Figure 4. Figure 4 reveals that the various estimated Δ*K_eff_* versus *da*/*dN* curves have not fully collapsed onto a single *R*-ratio-independent curve. However, it is reasonable to expect that, at *R* = 0.95, there should be minimal crack closure so that these curves should be expected to collapse onto the *R* = 0.95 Δ*K_eff_* versus *da*/*dN* curve. The corresponding Δ*K_eff_* versus *da*/*dN* curves obtained using Equation (5), i.e., Simple Scaling, are shown in Figure 5, where we see that these curves are now tightly grouped. The values of the function *U*(*R*) (= Δ*K_eff,da_*_/*dN*_/Δ*K_da_*_/*dN*_(*R*)) used in Figure 5 to estimate the closure-free curve are given in Table 2. Table 2 also contains the value of *da*/*dN* at which Equation (5) made the various curves coincide.

An important feature that is rarely commented on and is aptly illustrated in Figure 4 and Figure 5 is that Elber’s crack closure formulation would appear to only account for the effect of *R*-ratio in what is generally referred to as Regions I and II. This feature is also apparent in the Δ*K_eff_* versus *da*/*dN* curves given in [20] for the growth of long cracks in 4340 steel. This dataset is examined later in this paper.

## 5. Crack Growth in STOA TI-6AL-4V

**Figure 6:**
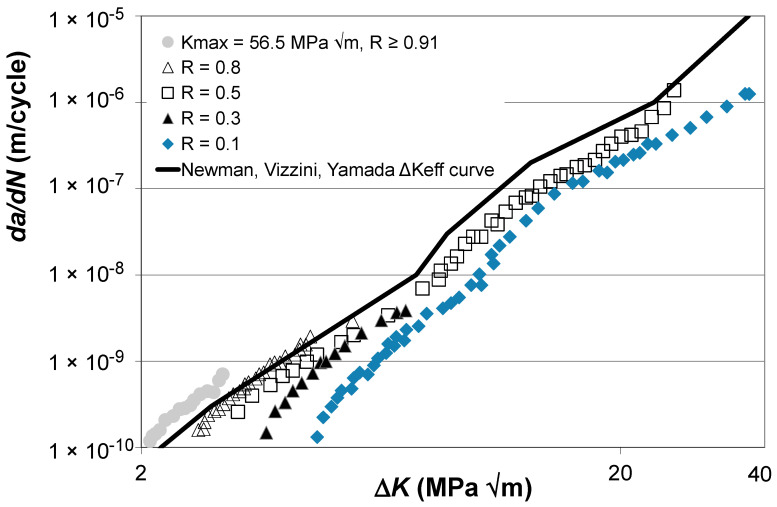
The Δ*K* versus *da*/*dN* curves given in [33] for STOA Ti-6Al-4V and the corresponding Δ*K_eff_* versus *da*/*dN* curve given in [34] (These curves are adapted from [33,34]).

Let us next examine the *R* = 0.8, 0.5, 0.3, and 0.1 Δ*K* versus *da*/*dN* curves given by Boyce and Ritchie [33] for STOA Ti-6Al-4V and the curve given in [33] for a high *K_max_* test, i.e., *K_max_* = 56.5 MPa √m, for which *R* ≥ 0.91 and hence can be expected to essentially be closure free. These curves are shown in Figure 6 along with the Δ*K_eff_* versus *da*/*dN* curve given in [34], which was obtained using the “analytical” crack closure equation [10] with a constraint factor of 2. Here, see that the Δ*K_eff_* versus *da*/*dN* curve given in [34] underestimates the crack closure-free curve, i.e., the *K_max_* = 56.5 MPa √m curve given in [33].

**Figure 7:**
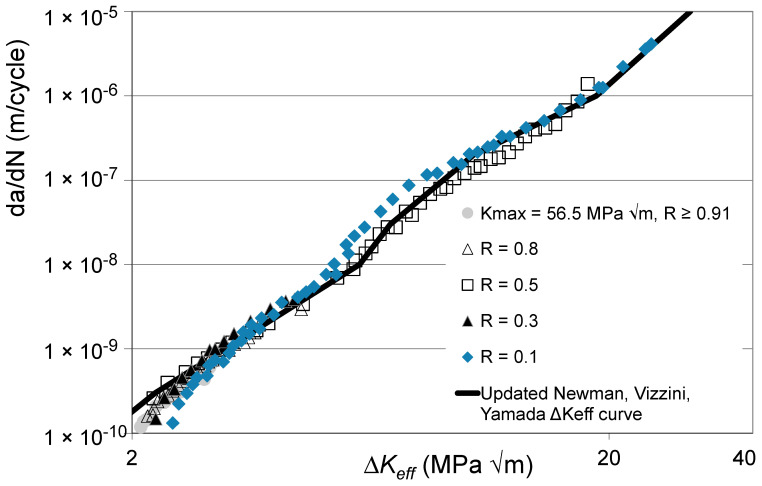
The Δ*K_eff_* versus *da*/*dN* curves obtained using Equation (5) and the corresponding updated Newman, Vizzini, Yamada curve given in [34] (The Newman, Vizzini, Yamada curve is adapted from [34]).

The closure-free Δ*K_eff_* versus *da*/*dN* curves determined using Equation (5) and the “updated” estimate of the Δ*K_eff_* versus *da*/*dN* curve given in [34], which was obtained by applying Equation (5) to the Δ*K_eff_* versus *da*/*dN* curve given in [34] and shown in Figure 6, are shown in Figure 7. Here, we again see that making use of Equation (5) not only collapses the various Δ*K_eff_* versus *da*/*dN* curves onto what is essentially a single curve, but it also enables the Δ*K_eff_* versus *da*/*dN* curve given in [34] to be collapsed onto what is essentially the same curve. The values of the function *U*(*R*) (= Δ*K_eff,da_*_/*dN*_/Δ*K_da_*_/*dN*_(*R*)) used in Figure 7 are given in Table 2.

## 6. Crack Growth in 7075-T7351

**Figure 8:**
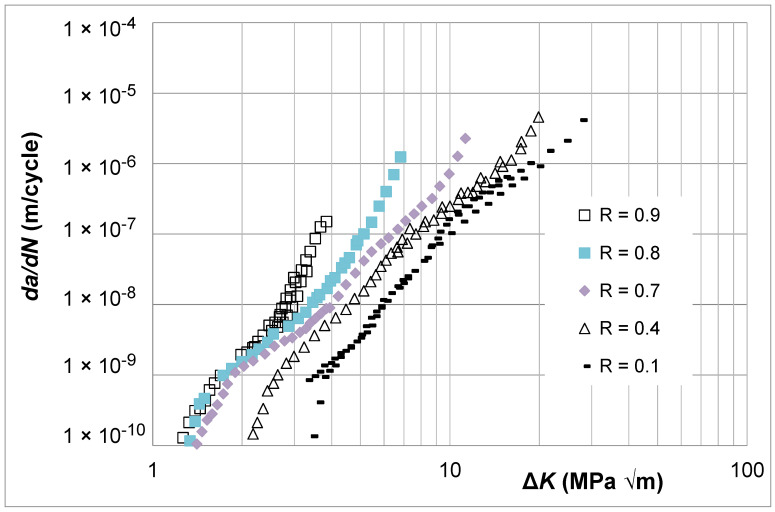
The Δ*K* versus *da*/*dN* curves for 7075-T7351 (These curves are adapted from [35]).

Consider the *R* = 0.1, 0.4, 0.7, 0.8, and 0.9 Δ*K* versus *da*/*dN* curves and the Δ*K_eff_* versus *da*/*dN* curve given in [35] for aluminum alloy 7075-T7351. These curves, which are shown in Figure 8, were obtained following the test protocols outlined in Sections 8.5 and 8.6 of ASTM E647-23b [1].

**Figure 9:**
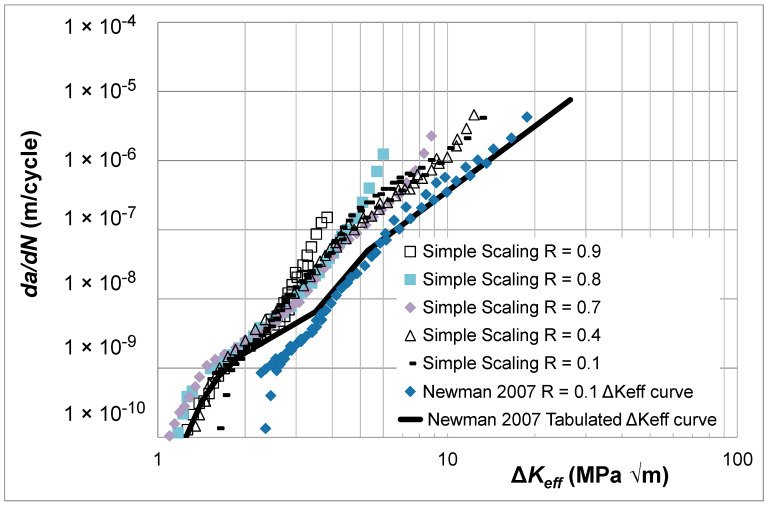
The Δ*K_eff_* versus *da*/*dN* curves obtained using Equation (5), i.e., Simple Scaling, the Δ*K_eff_* curve given in [35] that was determined from the *R* = 0.1 data, and the tabulated *da*/*dN* versus Δ*K_eff_* curve given in [35] (The *da*/*dN* versus Δ*K_eff_* curves are adapted from [33,39]).

The corresponding Δ*K_eff_* versus *da*/*dN* curves determined using Equation (5), i.e., using Simple Scaling, are shown in Figure 9. Here, we see that in comparison to the Δ*K_eff_* versus *da*/*dN* curves given in [35], these curves are now tightly grouped. To illustrate this, Figure 9 also contains plots of the Δ*K_eff_* versus *da*/*dN* curves given in [35] that were determined from the *R* = 0.1 and *R* = 0.9 curves shown in Figure 8. The values of the function *U*(*R*) (=Δ*K_eff,da_*_/*dN*_/Δ*K_da_*_/*dN*_(*R*)) used in Figure 9 are given in Table 2.

Figure 9 also contains a plot of the tabulated *da*/*dN* versus Δ*K_eff_* curve given in [35]. Here, we see that as a result of the relatively poor estimate of the *R* = 0.1 Δ*K_eff_* versus *da*/*dN* curve, which differs significantly from the *R* = 0.9 curve, the tabulated curve starts off (at low values of *da*/*dN*) with values that are close to that of the *R* = 0.9 curve and veers toward the *R* = 0.1 Δ*K_eff_* versus *da*/*dN* curve as the value of *da*/*dN* increases. As such, as the value of *da*/*dN* increases, this tabulated curve underestimates both the Δ*K_eff_* versus *da*/*dN* curve obtained using Equation (5) and the *R* = 0.9 Δ*K* versus *da*/*dN* curve.

## 7. Crack Growth in Aluminum Alloy 2324-T39

**Figure 10:**
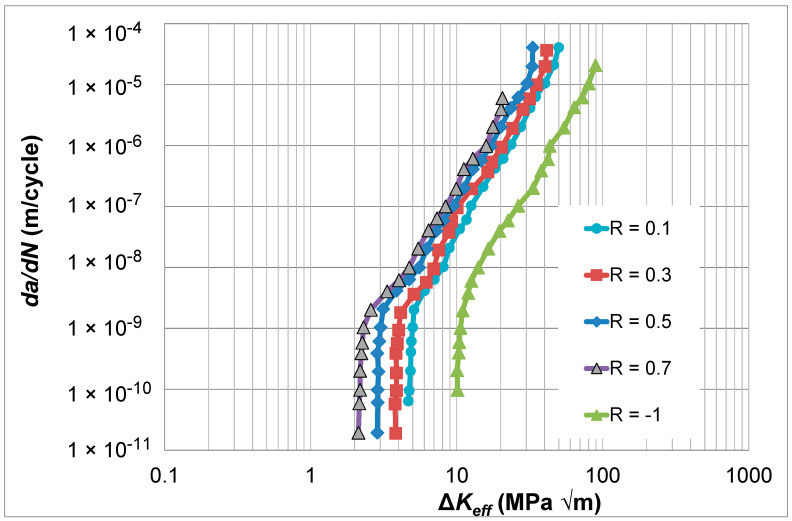
The *R* = 0.7, 0.5, 0.3, 0.1, and −1.0 Δ*K* versus *da*/*dN* curves for 2324-T39 (These curves are adapted from [36]).

**Figure 11:**
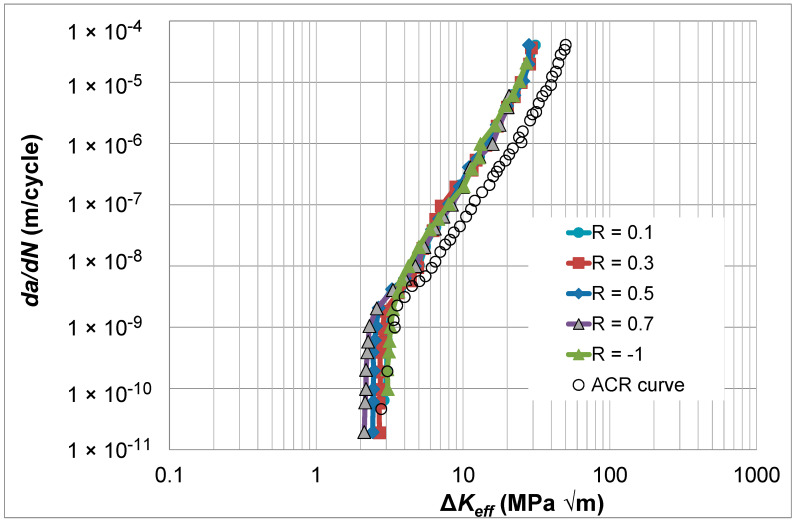
The *R* = 0.7, 0.5, 0.3, 0.1, and −1.0 Δ*K_eff_* versus *da*/*dN* curves obtained using Simple Scaling, i.e., Equation (5), and the corresponding ACR curve given in [36] for 2324-T39, which is adapted from [36].

As previously mentioned, ASTM E647-23b also suggests using what is termed the adjusted compliance ratio (ACR) test procedure, which was first developed by Donald et al. [15], to determine the Δ*K_eff_* versus *da*/*dN* curves. To investigate how the Simple Scaling formulation can be used in conjunction with this approach, let us consider the *R* = 0.7, 0.5, 0.3, 0.1, and -1.0 Δ*K* versus *da*/*dN* curves given in [36] for aluminum alloy 2324-T39. These curves, which are shown in Figure 10, were obtained using the test protocol outlined in Sections 8.5 and 8.6 of ASTM E647-23b [1]. This study found that the ACR-corrected curves did not collapse onto a single cure. This can be seen in Figure 11, which presents the ACR-corrected *R* = 0.1 and 0.7 *da*/*dN* versus Δ*K_eff_* curves. (Here, it should be noted that in [36], the *R* = 0.7 Δ*K_eff_* versus *da*/*dN* curve coincided with the *R* = 0.7 *da*/*dN* versus Δ*K* curve). As a result, ref. [36] suggested expressing *da*/*dN* as a function of both Δ*K_eff_* and *K_max_*. Figure 11 also presents the *da*/*dN* versus Δ*K_eff_* curves determined using Simple Scaling, i.e., Equation (5). As can also be seen in Figure 11, the need to express *da*/*dN* as a function of both Δ*K_eff_* and *K_max_* vanishes if Equation (5) is used to determine the various *da*/*dN* versus Δ*K_eff_* curves. The values of the function *U*(*R*) (=Δ*K_eff,da_*_/*dN*_/Δ*K_da_*_/*dN*_(*R*)) used in Figure 11 are given in Table 2.

## 8. Crack Growth in Aluminum Alloy 7249-T6511

**Figure 12:**
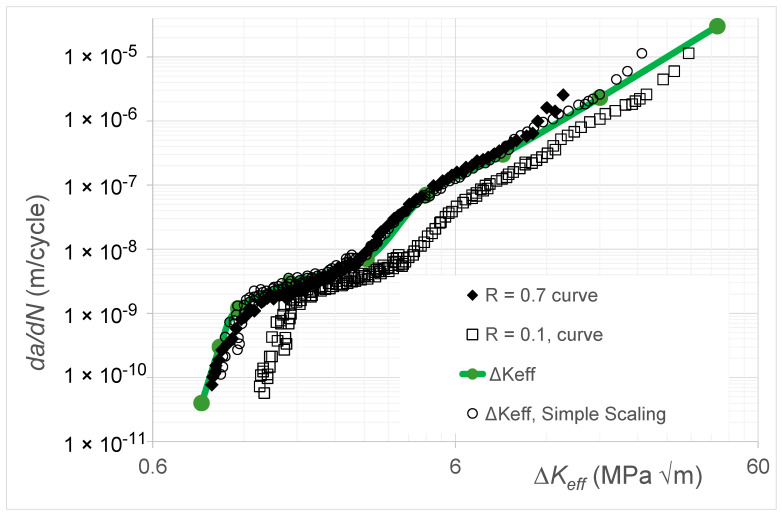
The *R* = 0.7 and 0.1 Δ*K* versus *da*/*dN* curves, the Δ*K_eff_* versus *da*/*dN* curve given in [37], and the Δ*K_eff_* versus *da*/*dN* curve obtained using Simple Scaling for aluminum alloy 7249-T6511 (The *da*/*dN* versus Δ*K_eff_* curve and the *da*/*dN* versus Δ*K* curves are adapted from [37]).

Let us next consider crack growth in aluminum alloy 7249-T6511. The *R* = 0.7 and 0.1 Δ*K* versus *da*/*dN* curves and the corresponding Δ*K_eff_* versus *da*/*dN* curve given in [37] are shown in Figure 12. This study reported that, for *da*/*dN* ≤ 10^−8^ m/cycle, compression pre-cracking constant amplitude (CPCA) and compression pre-cracking load reduction (CPLR) methods were used to generate the Δ*K* versus *da*/*dN* curves. A back face strain gauge was used to monitor both the crack length and the crack opening. The Δ*K_eff_* versus *da*/*dN* curves were determined using the “analytical crack closure equation” [10], with the constraint factor set to 1.8.

Figure 12 also contains the Δ*K_eff_* versus *da*/*dN* curve obtained using Equation (2), i.e., using the Simple Scaling approach. Here, we again see that Equation (5) yields a Δ*K_eff_* versus *da*/*dN* curve that is in good agreement with that given in [37]. The values of the function *U*(*R*) (=Δ*K_eff,da_*_/*dN*_/Δ*K_da_*_/*dN*_(*R*)) used in Figure 12 are given in Table 2.

## 9. Crack Growth in Mg AZ91E

**Figure 13:**
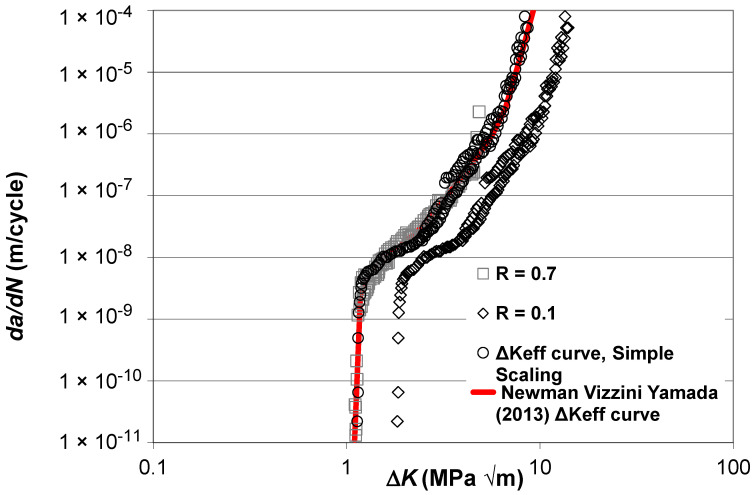
The *R* = 0.1 and 0.7 Δ*K* versus *da*/*dN* curves, the *da*/*dN* versus Δ*K_eff_* curve obtained using Equation (5), and the Δ*K_eff_* versus *da*/*dN* curve given in [34] for magnesium alloy Mg AZ91E (The *da*/*dN* versus Δ*K_eff_* curve and the *da*/*dN* versus Δ*K* curves and are adapted from [34]).

Let us next consider the *R* = 0.1 and 0.7 Δ*K* versus *da*/*dN* curves and the Δ*K_eff_* versus *da*/*dN* curves given in [34] for magnesium alloy Mg AZ91E, which is used in helicopters. (The heat treatment was not given in [34]). These curves are shown in Figure 13 along with the *da*/*dN* versus Δ*K_eff_* curve determined using Equation (5). As can be seen in Figure 13, the *da*/*dN* versus Δ*K_eff_* curve given in [34] and the curve estimated using Equation (5) are in good agreement. The values of the function *U*(*R*) (=Δ*K_eff,da_*_/*dN*_/Δ*K_da_*_/*dN*_(*R*)) used in Figure 13 are given in Table 2.

## 10. Crack Growth in 4340 Steel

**Figure 14:**
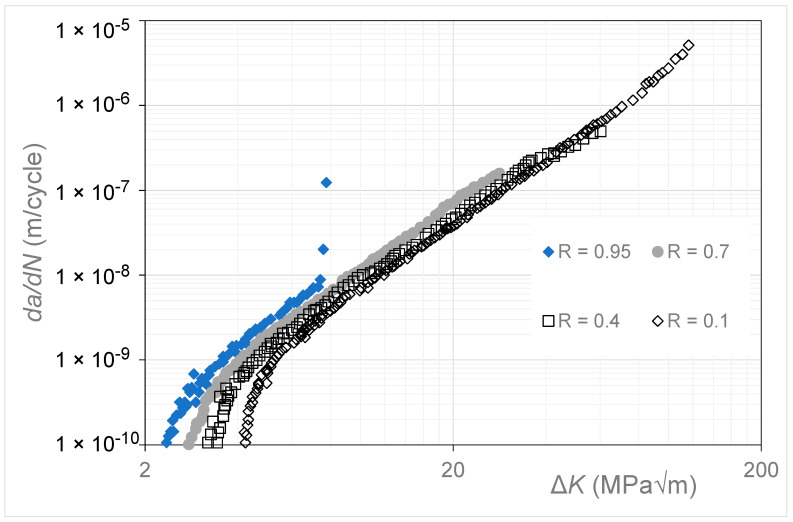
The *R* = 0.95, 0.7, 0.4, and 0.1 Δ*K* versus *da*/*dN* curves for 4340 steel (The *da*/*dN* versus Δ*K* curves are adapted from [20]).

**Figure 15:**
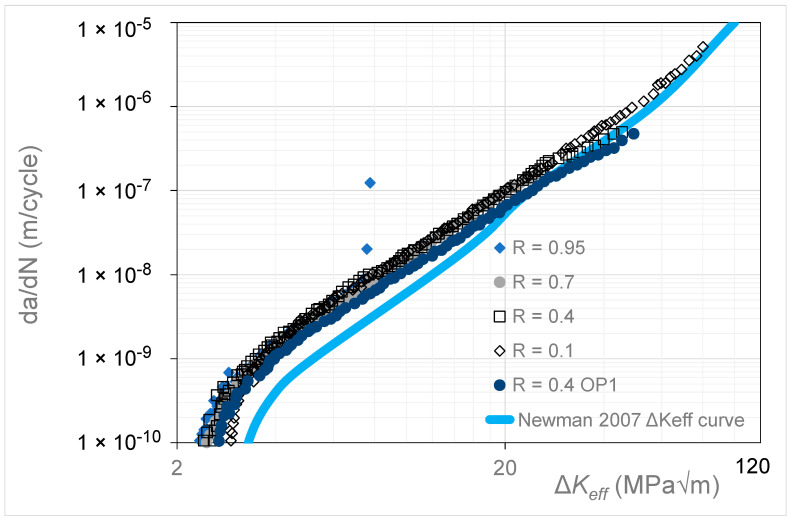
The Δ*K_eff_* versus *da*/*dN* curves obtained using Equation (5) (Simple Scaling) and the corresponding curves given in Newman 2007 [20] for 4340 steel (The *da*/*dN* versus Δ*K_eff_* curve is adapted from [20]).

Figure 14 presents the *R* = 0.95, 0.7, 0.4, and 0.1 Δ*K* versus *da*/*dN* curves given in [20] for high-strength steel 4340. This study reported that these tests used a back face strain gauge to monitor both the crack length and the crack opening force, and that, for *da*/*dN* ≤ 10^−8^ m/cycle, compression pre-cracking constant amplitude (CPCA) and load reduction (CPLR) methods were used to generate the Δ*K* versus *da*/*dN* curves. Figure 15 presents the corresponding *R* = 0.95, 0.7, 0.4, and 0.1 *da*/*dN* versus Δ*K_eff_* curves obtained using Equation (5), as well as the curve obtained in [34] using the ASTM E647 crack opening test protocol for the *R* = 0.4 test. This curve is labeled *R* = 0.4 OP1. Figure 15 also contains the Δ*K_eff_* versus *da*/*dN* curve given in [35] for crack growth in this material. In this (latter) instance, i.e., in [35], the tests studied were limited to *R*-ratios that were less than or equal to 0.7. (In this context, it should be noted that, in [20], the *R* = 0.95 Δ*K_eff_* versus *da*/*dN* curve coincided with the *R* = 0.95 Δ*K* versus *da*/*dN* curve).

Figure 15 reveals that Equation (5) yields curves that are in good agreement with the high *R*-ratio tests. The exception to this observation is the Δ*K_eff_* versus *da*/*dN* curve given in [20] and referred to in Figure 15 as the “Newman 2007 Δ*K_eff_* versus *da*/*dN* curve”. This latter curve underestimates both the *R* = 0.95 and the *R* = 0.4 OP1 curves and would thus appear to be erroneous/invalid. The values of the function *U*(*R*) (= Δ*K_eff,da_*_/*dN*_/Δ*K_da_*_/*dN*_(*R*)) used in Figure 15 are given in Table 2.

## 11. Crack Growth in Mn-Cr Austenitic Steel

**Figure 16:**
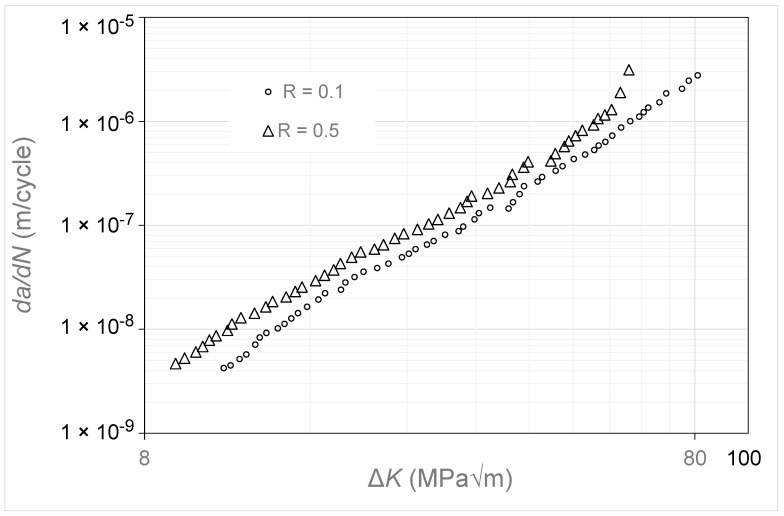
The *R* = 0.1 and 0.5 Δ*K* versus *da*/*dN* curves for Mn-Cr (The *da*/*dN* versus Δ*K* curves are adapted from [38]).

**Figure 17:**
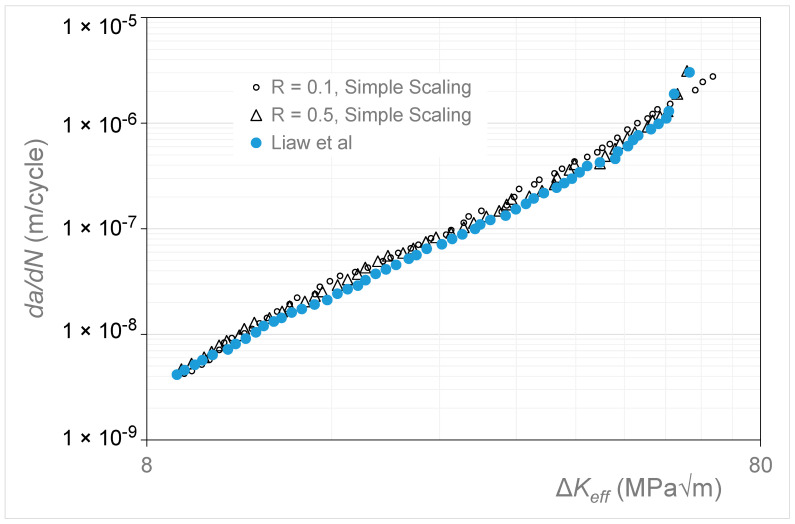
The Δ*K_eff_* versus *da*/*dN* curves obtained for Mn-Cr and the corresponding curve given in [38].

Let us next consider the crack growth data presented in [38] for Mn-Cr austenitic steel. The *R* = 0.5 and 0.1 Δ*K* versus *da*/*dN* curves are shown in Figure 16. Figure 17 presents the Δ*K_eff_* versus *da*/*dN* curve given in [38], which was obtained from the *R* = 0.5 curve using what was termed an “unloading elastic compliance technique”—see [38] for details—along with the Δ*K_eff_* versus *da*/*dN* curve obtained using the Simple Scaling approach. (Here, it should be noted that, in [38], the *R* = 0.5 Δ*K_eff_* versus *da*/*dN* curve essentially coincided with the *R* = 0.5 Δ*K* versus *da*/*dN* curve. Consequently, in the Simple Scaling approach, the *R* = 0.1 curve was scaled so as to match the *R* = 0.5 curve at a crack growth rate (*da*/*dN*) of approximately 10^−8^ m/cycle). As can be seen in Figure 17, Equation (5) yields an Δ*K_eff_* versus *da*/*dN* curve that is in good agreement with that given in [38]. The values of the function *U*(*R*) (=Δ*K_eff,da_*_/*dN*_/Δ*K_da_*_/*dN*_(*R*)) used in Figure 17 are given in Table 2.

## 12. Crack Growth in Rene 95

**Figure 18:**
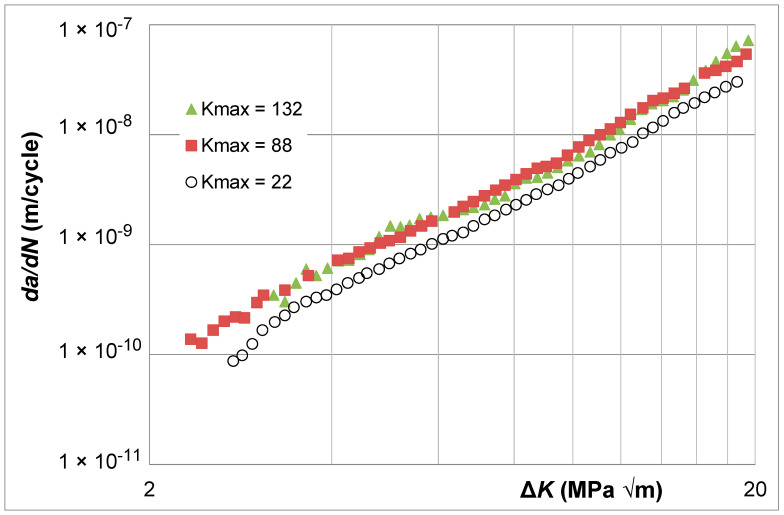
The Δ*K_eff_* versus *da*/*dN* curves for Rene 95 (The *da*/*dN* versus Δ*K* curves are adapted from [39]).

**Figure 19:**
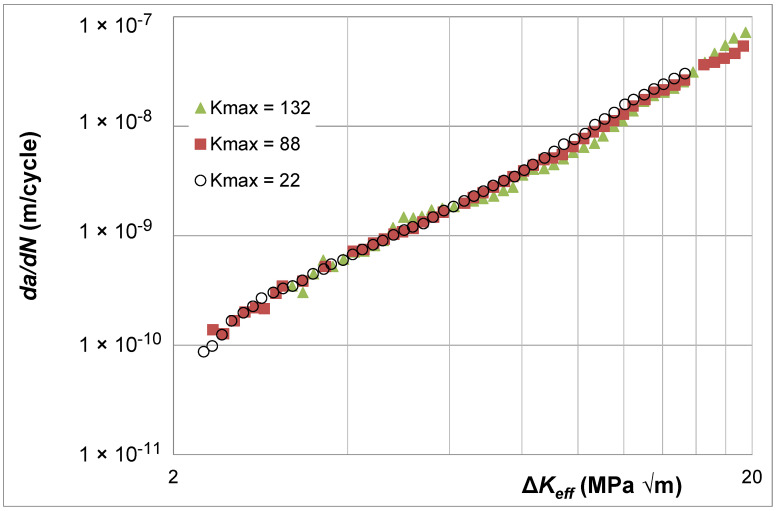
The Δ*K_eff_* versus *da*/*dN* curves determined using Equation (5) for the data shown in Figure 18.

Let us finally consider the Δ*K* versus *da*/*dN* curves given in [39] for super alloy Rene 95. These were *K_max_* tests with *K_max_* values of 22, 88, and 132 MPa √m. The Δ*K* versus *da*/*dN* curves are shown in Figure 18. Here, we see that the Δ*K* versus *da*/*dN* curves associated with *K_max_* = 88 MPa √m and 132 MPa √m essentially coincide, which suggests that these curves essentially represent (are close to) the Δ*K_eff_* versus *da*/*dN* curve for Rene 95. Figure 19 illustrates that using Simple Scaling collapses the *K_max_* = 22 MPa √m curve onto the same curve. The values of the function *U*(*R*) (=Δ*K_eff,da_*_/*dN*_/Δ*K_da_*_/*dN*_(*R*)) used in Figure 19 are listed in Table 2.

**Table 2:** Values of the function *U*(*R*) used in Figure 5, Figure 7, Figure 9, Figure 11, Figure 12, Figure 13, Figure 15, Figure 17 and Figure 19.

Material	*R*-Ratio	*U*(*R*) = Δ*K_eff,da_*_/*dN*_/Δ*K_da_*_/*dN*_(*R*)	Value of da/dN (m/Cycle) at Which the Curves Were Matched
9310 steel	0.95	1.00	2 × 10^−9^
	0.9	0.960	2 × 10^−9^
	0.7	0.829	2 × 10^−9^
	0.4	0.791	2 × 10^−9^
	0.1	0.713	2 × 10^−9^
STOA Ti-6Al-4V	0.8	0.820	10^−9^
	0.5	0.697	10^−9^
	0.3	0.615	10^−9^
	0.1	0.523	10^−9^
	*K_max_* = 51.6 MPa √m	1.00	10^−9^
	Δ*K_eff_* curve in [34]	0.8	10^−9^
7075-T7351	0.9	1.00	2 × 10^−9^
	0.8	0.88	2 × 10^−9^
	0.7	0.78	2 × 10^−9^
	0.4	0.62	2 × 10^−9^
	0.1	0.47	2 × 10^−9^
2324-T39	0.7	1.00	10^−8^
	0.5	0.848	10^−8^
	0.3	0.707	10^−8^
	0.1	0.624	10^−8^
	−1	0.303	10^−8^
7249-T6511	0.7	1.00	10^−8^
	0.1	0.70	10^−8^
4340 steel	0.95	1.00	10^−8^
	0.7	0.889	10^−8^
	0.4	0.755	10^−8^
	0.1	0.750	10^−8^
	0.4 OP1	0.878	10^−8^
Mg AZ91E	0.7	1.00	10^−8^
	0.1	0.620	10^−8^
Mn-Cr Austenitic steel	0.7	1.0	10^−8^
	0.1	0.826	10^−8^
Rene 95	*K_max_* = 132 MPa √m	1.0	10^−9^
	*K_max_* = 88 MPa √m	1.0	10^−9^
	*K_max_* = 22 MPa √m	0.82	10^−9^

## 13. Discussion

**Figure 20:**
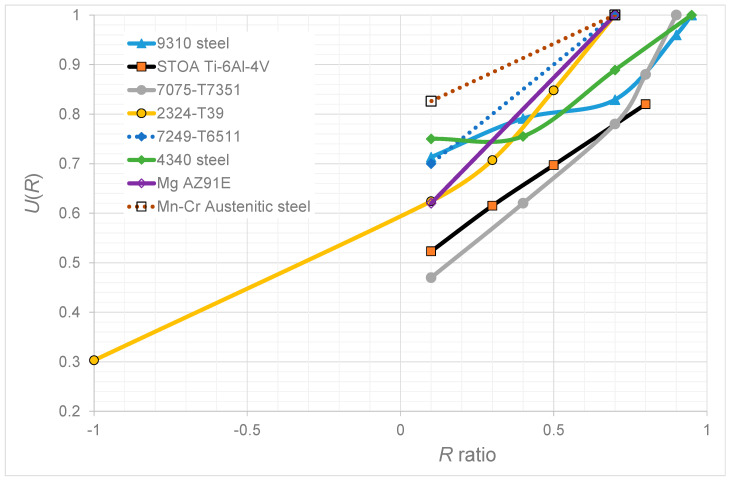
The *U*(*R*) versus *R* curves obtained in this study.

A plot of the relationship between *U*(*R*) and R determined in each of the various studies is given in Figure 20. Unfortunately, there does not appear to be any discernable trend. Nevertheless, the current paper, when taken together with the numerous examples presented in [22,23,24], would appear to support the hypothesis that for many conventionally manufactured metals, *R*-ratio effects are, to a first approximation, merely a reflection of the effect of mean stress on the fatigue threshold. However, it should be noted that this observation does not hold for additively manufactured (AM) metals. Indeed, it is now known [40,41,42] that, for a given AM material, crack growth is a function of both the fatigue threshold and the cyclic fracture toughness. It is also known [40,41,42] that there are numerous instances when both the damage tolerance and the durability of an AM material can be modeled using the governing crack growth equation determined for its conventionally manufactured counterpart and merely making allowances for the changes in the fatigue threshold and the cyclic fracture toughness terms that are associated with the particular build process and the orientation of the crack to the build direction. Unfortunately, there is currently no explanation for this observation. Nevertheless, the authors suspect that the explanation for the current observation (for conventionally manufactured materials) and the observation referred to above for AM materials are linked. The challenge is to discover the fundamental physics behind these various observations.

## 14. Conclusions

This paper has attempted to illustrate how the Simple Scaling formula for Elber’s crack closure function *U*(*R*), i.e., Equation (5), is a useful addition to the tools available for assessing crack growth. However, it should be stressed that, whilst this approach has been shown to help for nine different materials, it may not always be applicable. In other words, it is merely a tool that may be useful for assessing fatigue crack growth.

In this context, the present paper has shown how Simple Scaling is able to determine the closure-free Δ*K_eff_* versus *da*/*dN* curves for nine different conventionally manufactured metals. The present paper also illustrates its use when studying the effects of both the test temperature and *R*-ratio on crack growth in titanium alloy Ti-6Al-4V ELI. In this instance, we see that when *da*/*dN* is plotted against Δ*K*/Δ*K_th_*, then, to a first approximation, all of the different curves now essentially collapse onto a curve that is independent of both the *R*-ratio and the test temperature.

As aptly illustrated in [23,24,25,26], this formulation has potential applications outside of assessing the closure-free crack growth curve. Indeed, it has previously been shown to be useful in identifying the fracture mechanics parameters that govern the variability in the growth of cracks in materials ranging from medium-entropy alloys to polymers and the delamination growth in composites with different levels of pre-cracking.

As such, the Simple Scaling variant of Elber’s original hypothesis would appear to have the advantage over other approaches in that it can help to clarify the important parameters for a wide cross-section of problems. It also has the potential to tackle problems where, whilst the *R*-ratio is fixed, the test conditions are different, for example, different test temperatures, different levels of irradiation, etc. The ability of other approaches to perform this task for tests where the *R*-ratio is fixed and the question is “what is the effect of different test temperatures or different levels of irradiation?” is somewhat limited.

A feature of this Simple Scaling approach to assessing the closure-free Δ*K_eff_* versus *da*/*dN* curve is that it is consistent with Elber’s original hypothesis, that it can be quickly and easily performed, and that there are instances, for example when the estimates for the Δ*K_eff_* versus *da*/*dN* curves obtained by other approaches do not coincide sufficiently, where it may help to further collapse these curves onto what is essentially a single Δ*K_eff_* versus *da*/*dN* curve.

## Figures and Tables

**Table 1:** The values of Δ*K*_th_ used in Figure 2.

*R*-Ratio and Test Temperature	Δ*K*_th_ (MPa √m)
*R* = 0.5, 25 °C	4.8
*R* = 0.06, 25 °C	6.4
*R* = 0.06, 250 °C	5.8
*R* = −1, 25 °C	12.5
*R* = −1, 250 °C	11.1
*R* = 0.1, RT. 20 Hz	4.9
*R* = 0.1, RT	6.0
*R* = 0.1, 4 °K, Test 1	5.9
*R* = 0.1, 4 °K, Test 2	5.1

## Data Availability

The data will be made available at the completion of the project.
